# Advance Progress in Assembly Mechanisms of Carrier-Free Nanodrugs for Cancer Treatment

**DOI:** 10.3390/molecules28207065

**Published:** 2023-10-13

**Authors:** Xiaoyu Zhang, Shuyang Hu, Lifei Huang, Xiyue Chen, Xin Wang, Ya-nan Fu, Hui Sun, Guofeng Li, Xing Wang

**Affiliations:** 1 State Key Laboratory of Organic-Inorganic Composites, Beijing Laboratory of Biomedical Materials, Beijing University of Chemical Technology, Beijing 100029, China; 2 Department of Hepatology, Tongliao Infectious Disease Hospital, Tongliao 028000, China; 3 Department of Interventional Ultrasound, PLA Medical College & Fifth Medical Center of Chinese PLA General Hospital, Beijing 100039, China

**Keywords:** cancer treatment, carrier-free nanodrugs, noncovalent interactions, covalent bonds, metal ions coordination

## Abstract

Nanocarriers have been widely studied and applied in the field of cancer treatment. However, conventional nanocarriers still suffer from complicated preparation processes, low drug loading, and potential toxicity of carriers themselves. To tackle the hindrance, carrier-free nanodrugs with biological activity have received increasing attention in cancer therapy. Extensive efforts have been made to exploit new self-assembly methods and mechanisms to expand the scope of carrier-free nanodrugs with enhanced therapeutic performance. In this review, we summarize the advanced progress and applications of carrier-free nanodrugs based on different types of assembly mechanisms and strategies, which involved noncovalent interactions, a combination of covalent bonds and noncovalent interactions, and metal ions-coordinated self-assembly. These carrier-free nanodrugs are introduced in detail according to their assembly and antitumor applications. Finally, the prospects and existing challenges of carrier-free nanodrugs in future development and clinical application are discussed. We hope that this comprehensive review will provide new insights into the rational design of more effective carrier-free nanodrug systems and advancing clinical cancer and other diseases (e.g., bacterial infections) infection treatment.

## 1. Introduction

Cancer possesses has a high global incidence rate and mortality, which is extremely harmful to human life and health [1]. Traditional chemotherapy (CT) relying on anticancer drugs is the most utilized clinical therapeutic modality [2,3]. However, anticancer drugs can only achieve limited success, they still face challenges including poor solubility, low bioavailability, severely toxic side effects, insufficient stability, and rapid blood clearance, which pose grievous barriers to cancer treatment [4]. To address the aforementioned challenges, numerous innovative and elaborately designed therapeutic drug delivery technologies have been devoted to improving cancer therapy efficiency.

With the development of nanotechnology, nanocarrier-based drug delivery systems (DDS) offer advanced opportunities for cancer diagnosis and therapy [5,6]. In general, traditional nanocarriers include polymer micelles [7], dendrimers [8], liposomes [9] and inorganic nanoparticles [10]. These nanocarriers can significantly increase stability, improve biocompatibility, prolong blood circulation, and enhance tumor accumulation and the therapeutic efficacy of the encapsulated active drugs [11,12], which distinctly facilitate the development of nanomedicines. Nevertheless, the relevant nanocarrier-based DDS still face some common challenges, such as complicated preparation or purification processes, lack of active functionality, low drug loading capacity and incomplete drug release, as well as the potential systemic toxicity of carriers-themselves, which hinder the scaled-up production and practical use of nanocarrier-based nanomedicines [13,14]. Rational designing advanced nano-DDSs for efficient cancer and other related disease therapy still remains challenging.

Carrier-free nanodrugs emerge as smart nanodrug delivery platforms for cancer treatment. Commonly, carrier-free nanodrugs are fabricated via the self-assembly or co-assembly of several drug active ingredients without or with little use of inert materials, in which the active ingredients are mainly drugs with different therapeutic mechanisms [15], including chemotherapeutic drugs [16], photosensitizers [17], targeting agents [18], antibodies to certain proteins [19], genes [20], and other functional biomolecules. This endows carrier-free nanodrugs with desirable advantages over nanocarrier-based DDS: (ⅰ) simple and green assembly process; (ⅱ) integration of nano-based diagnosis and therapy; (ⅲ) inhibition of multidrug resistance (MDR); (ⅳ) synergistic therapeutic effects superior to multidrug combinations with equal-dose; (ⅴ) high drug loading capacity; (ⅵ) low toxicity and good pharmacokinetics [21,22,23]. Therefore, carrier-free nanodrugs can compensate for the shortcomings of traditional nanocarriers, achieving efficient drug utilization and synergistic therapy effects.

Based on the above distinct benefits, a series of assembly mechanisms and therapeutic strategies have been proposed to achieve better therapeutic outcomes. Currently, the assembly mechanisms and strategies during the preparation of carrier-free nanodrugs are mainly classified into the following categories. First, drug molecules or functional biomolecules with special physical properties, chemical structure, and aggregation behavior automatically organize themselves into structurally well-defined nanodrugs driven by noncovalent interactions. These noncovalent interactions consist of hydrophobic interaction, hydrogen bonding interaction, electrostatic interaction, and π-π stacking interaction, etc. [24,25,26]. During the assembly process, the driving force is a key factor affecting the success of carrier-free nanodrugs. Second, covalent bonds are employed to combine one or multiple drug molecules or drug molecules with functional biomolecules to form prodrugs, which are subsequently self-assembled to construct carrier-free nanodrugs through noncovalent interactions [27]. The prodrugs usually display certain special responsive structures, which can achieve controlled release of nanodrugs at specific sites. For example, pH-responsive imine (Schiff-base) bonds [28], reduction-responsive disulfide bonds [29], easily hydrolyzable ester bonds [16], and enzyme-cleavable specific peptide linkers [30]. Third, carrier-free nanodrugs can be also designed directly by coordination-driven self-assembly of metal ions with drug molecules or functional biomolecules [31,32]. Coordination covalent bonds exhibit stable and dynamic behavior in complex environments, and the strength is between weak noncovalent interactions and strong covalent bonds [33].

**Scheme 1:**
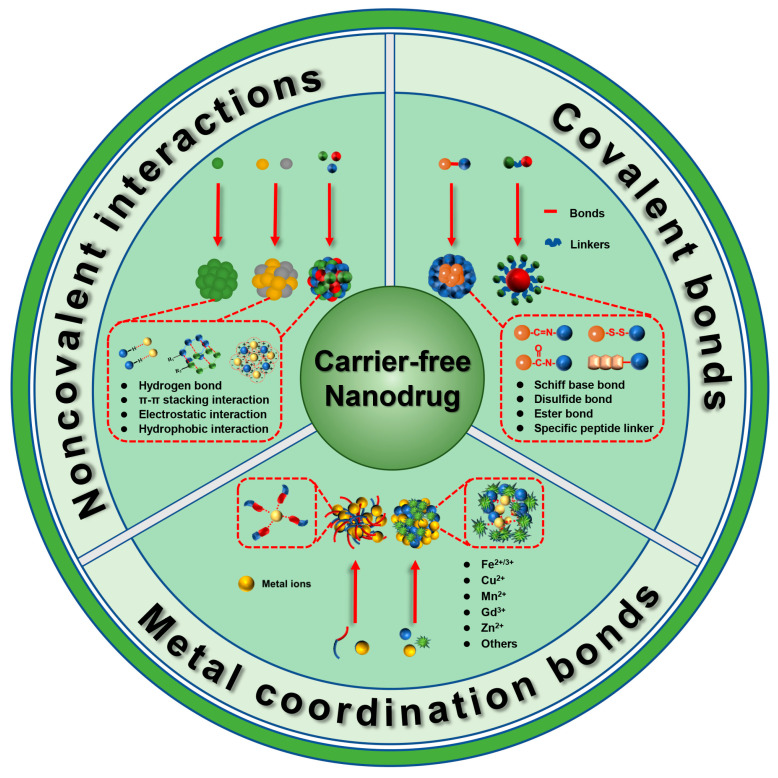
Schematic representation of the carrier-free nanodrugs based on various assembly mechanisms and strategies for cancer treatment.

Nowadays, many excellent published reviews have summarized the preparation methods, assembled ingredients, physicochemical properties, and external modifications [11,21,23,34,35,36]. The self-assembly mechanisms and strategies involved in their preparation are few systematically discussed. In this review, based on the assembly mechanism and strategy involved during carrier-free nanodrugs preparation, we summarize and discuss the research progress on the construction of carrier-free nanodrugs via noncovalent interactions, the combination of covalent bonds and noncovalent interactions, along with metal ions coordination-driven assembly (Scheme 1). Simultaneously, the relevant applications of various carrier-free nanodrugs for antitumor applications are also presented. Finally, we discuss the future prospects and challenges of carrier-free nanodrugs in clinical applications.

## 2. Noncovalent Interactions-Driven Carrier-Free Nanodrugs

Noncovalent forces play an important role in constructing carrier-free nanodrugs [37]. The special functional groups of active agents induce intermolecular noncovalent interactions between them or active agents and water molecules [38]. For example, aliphatic groups can cause hydrophobic interactions [39], hydroxyl/carboxyl groups interact via hydrogen bonds [40], aromatic groups can induce π-π stacking interactions [41], and ionic groups show electrostatic interactions [42]. Drug molecules and functional biomolecules can directly form carrier-free nanodrugs through these noncovalent interactions in the assembly medium solution [43,44,45], which effectively alleviates the problems of poor drug solubility, low anticancer efficiency, and serious side effects.

### 2.1. Hydrogen Bonding Interaction

Hydrogen bonds are an important noncovalent driving force for the self-assembly of carrier-free nanodrugs, which mainly exist between H atoms and electronegative atoms (e.g., O, F, and N) and are a relatively weak force [46]. Typically, a large number of drugs and functional biomolecules (e.g., anticancer drugs, photosensitizers, nucleic acids, etc.) with multiple hydrogen bonding sites, such as amide groups, amino groups, hydroxyl groups, or carboxyl groups can promote hydrogen bond-driven assembled nanostructures [47]. The assembly principle of hydrogen-bonded nanomedicine typically depends on the use of hydrogen bond and hydrophobic interactions working together, in which hydrogen bond-directed recognition occurs shielding the hydrophobic microenvironment in the medium solution [40]. Thus, one or more drugs and functional biomolecules can co-assemble to form carrier-free nanodrugs by hydrogen bonding and hydrophobic interactions during a single ordered process.

**Figure 1:**
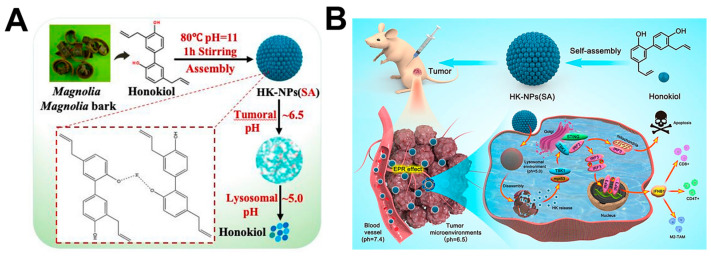
(**A**) Preparation of HK-NPS by intermolecular hydrogen bonding interactions. (**B**) HK-NPS for efficient immunotherapy of colorectal cancer with P53 mutations. Reprinted with permission from ref. [49]. Copyright 2022, American Chemical Society.

Natural honokiol (HK) is a *Magnolia* bar-derived anticancer drug for clinical trials in advanced non-small cell lung cancer [48]. However, HK shows hydrophobicity, low bioavailability and rapid metabolism, which greatly limit its application. Ji et al. employed HK to self-assemble into carrier-free nanoparticles (SA) via intermolecular hydrogen bonds and hydrophobic interactions (Figure 1A) [49]. During the assemble process, hydrogen bonding interactions drove 1D complex units’ formation, while hydrophobic units promoted further 3D self-assembly, thereby forming SA with hydrophilic outward and hydrophobic inward, respectively. SA exhibited good solubility and stability, excellent tumor-targeting ability and antitumor immunity (Figure 1B), which enhanced the targeted therapy of HK. This work indicates that hydrogen bonding and hydrophobic interactions-driven self-assembly offer a safe and “molecularly economical” strategy to rationally design clinical agents, which fully exploits the assembly concept of green chemistry.

Some natural small molecules (NSMs) usually contain chemical structures with hydrophobic skeletons and active groups, which can offer hydrogen bond sites. Therefore, the driving forces between two NSMs co-assembly mainly involve hydrogen bonding and hydrophobic interactions. Wang’s group has developed a variety of carrier-free nanodrugs using pure NSMs by hydrogen bonding and hydrophobic interactions [50]. The co-assembled NPs demonstrated strong synergistic antitumor effects and reliable biological safety. Dihydroartemisinin (DHA) is a plant-derived natural drug for effective cancer treatment. Unfortunately, the poor solubility and bioavailability of DHA seriously hamper its clinical application. Thus, Li et al. prepared carrier-free nanoparticles (DHA NPs) based on the hydrogen bonding and hydrophobic interactions between DHA molecules [51]. Compared with pure DHA molecules, DHA NPs not only overcame the poor water solubility of DHA, but also revealed favorable drug-loading, stability, acid-responsive drug release, and antitumor efficacy. All these studies demonstrate the importance of hydrogen bonding in the assembly process of carrier-free nanodrugs.

### 2.2. π-π Stacking Interaction

The π-π stacking interaction is a weak noncovalent interaction in chemistry, which usually coexists with hydrophobic interaction, particularly in cases involving aromatic groups with bonded structures [52,53]. This π-π stacking interaction typically preserves the functional attributes of the compounds and serves as a primary driving force for self-assembling drugs or functional biomolecules with high π-electron densities into carrier-free nanodrugs, showing potentially wide-ranging applications [54,55].

**Figure 2:**
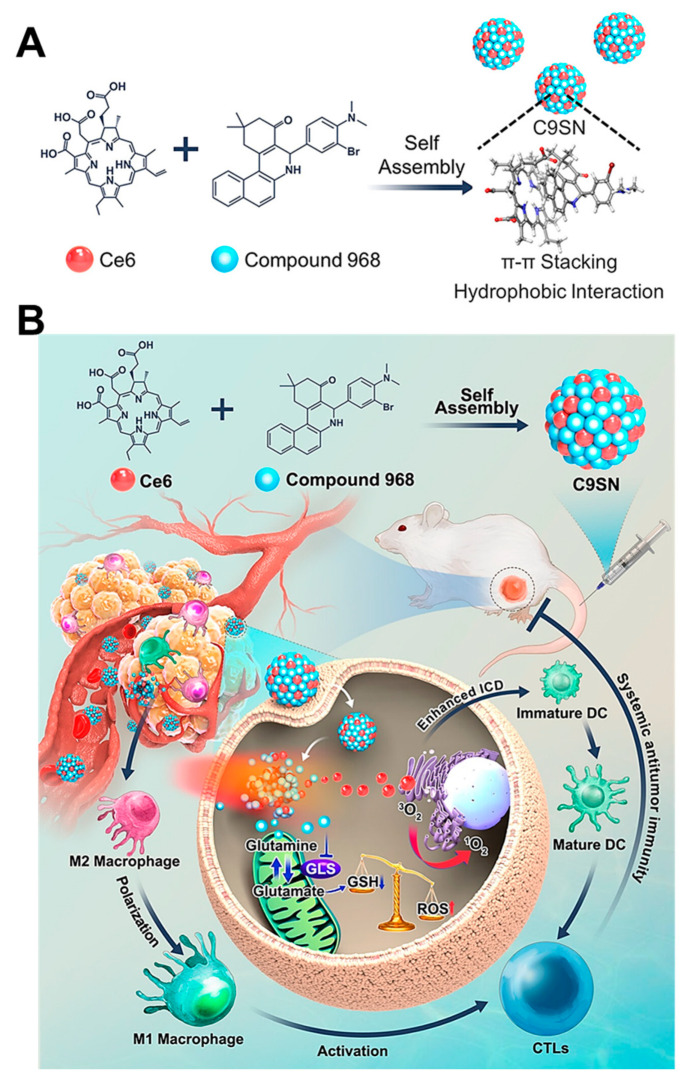
(**A**) Preparation of C9SN by π-π stacking and hydrophobic interactions. (**B**) C9SN and laser irradiation synergistically mediate anti-tumor immunotherapy. Reprinted with permission from ref. [60]. Copyright 2023, American Chemical Society.

Most photosensitizers have many aromatic π-conjugated structures with C–C double bonds and electron-rich heterocycles [56]. These structures play a pivotal role in facilitating the co-assembly of photosensitizers with active drug molecules via π-π interactions, which is beneficial for enhancing the cellular internalization and photodynamic efficiency of photosensitizers. Photosensitizer Chlorin e6 (Ce6) with a hydrophobic conjugated skeleton can interact with various hydrophobic chemotherapeutic agents [57]. Generally, photosensitizer loading in dense carrier materials possesses low loading capacity and quenching effect due to aggregation [58]. For example, Wang et al. constructed a Ce6-loaded ZIF-8@ssPDA nanosystem, which improved the bioavailability of Ce6 but still had the risk of Ce6 quenching due to the strong sealing of the carrier [59]. The carrier-free nanodrug delivery systems can better avoid this problem. To achieve synergistic effects with low toxicity, Mai and colleagues designed a carrier-free immunotherapeutic booster (C9SN) for the co-delivery of the glutaminase inhibitor compound 968 (C968) and Ce6 to tumor sites. This design harnessed dual synergistic mechanisms involving both π-π stacking and hydrophobic interactions between C968 and Ce6 molecules (Figure 2A) [60]. Ce6 is uniformly distributed inside C9SN to avoid the aggregation quenching effect. C9SN displayed high drug loading capacity enhanced cellular uptake, and improved tumor accumulation. This carrier-free C9SN triggered tumor immunogenicity and collaboratively reshaped the immunosuppressive tumor microenvironment under laser irradiation, which recruited and activated the cytotoxic T-lymphocytes, enhancing the systemic antitumor immunotherapy effect (Figure 2B).

Recently, the drug-drug delivery systems have been designed by employing π-π stacking interaction and hydrophobic interaction between two molecules. Wang et al. reported carrier-free nanoparticles (DiR-DOX NPs) co-assembled by doxorubicin (DOX) and the near-infrared photosensitizer DiR, further modified with a small amount of PEG (DiR-DOX-PEG NPs) [61]. The hydrophobic interaction of the alkyl chains and π-π stacking interaction between the conjugated structures of the two molecules drove the co-assembly process. Based on the combined action of chemotherapy and photothermal therapy, DiR-DOX-PEG NPs effectively eliminated tumors.

Multicomponent carrier-free nanodrugs involving multiple drugs or functional biomolecules can be also assembled by intermolecular π-π stacking and hydrophobic interactions. Qin’s team co-assembled Ce6, celecoxib (cyclooxygenase-2 inhibitor), and 6-thio-2′-deoxyguanosine (6-thio-dG, telomere-targeting drug) to form multicomponent carrier-free photodynamic nanodrugs (CC-6td NPs) through π-π stacking and hydrophobic interactions [62]. The CC-6td NPs could regulate dendritic cells (DCs) for robust immune responses, enhancing immunotherapeutic effects on tumors and inhibiting primary/abdominal tumor growth and postoperative recurrence. Furthermore, Wang et al. combined CDK4/6 protein hydrolysis-targeted chimeras (PROTAC) with Ce6 to form carrier-free nanoparticles via π-π stacking and hydrophobic interactions [63]. The dual-drugs-based nanoparticles improved antitumor efficacy and readjusted the immune-suppressing tumor microenvironment to enhance anti-tumor immunity.

### 2.3. Electrostatic Interaction

Electrostatic interaction, one noncovalent bond arising from opposite charges, mainly relies on the charge ratio, pH level, concentration, and ionic strength [64]. The strength of electrostatic interaction is notably influenced by the distance between charge centers and charge number, which directly impacts the interaction’s potency [65]. Therefore, drugs or functional biomolecules with charged groups, such as NH^3+^ and COO^−^ can self-assemble to form carrier-free nanodrugs via electrostatic forces.

**Figure 3:**
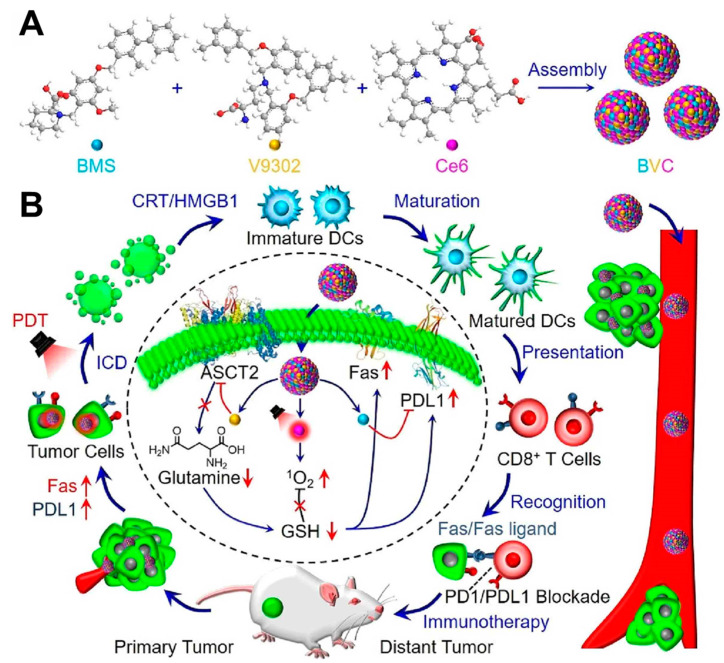
The preparation and proposed mechanism of BVC for photodynamic activated cascade immunotherapy through tumor metabolism reprogramming. (**A**) BVC preparation by self-assemble BMS, V9302 and Ce6. (**B**) BVC could passively accumulate at tumor site to inhibit ASCT2 and down-regulate glutamine and GSH, leading to the metabolism reprogramming of tumor cells to increase the expressions of Fas and PDL1. The elevated production of 1O2 could enhance PDT efficacy to eliminate primary tumor cells and induce ICD to release tumor associated antigens of CRT and HMGB1. Reprinted with permission from ref. [66]. Copyright 2023, Elsevier.

Creating ternary-component self-delivery nanodrugs that consider dosage, morphology, stability, and multi-synergistic mechanisms poses challenges. A combination of hydrophobic and electrostatic interactions between molecules can enhance drug physicochemical attributes during self-assembly. Zhao and coworkers prepared a carrier-free photodynamic immunostimulant (BVC) by integrating the Ce6, the ASCT2 inhibitor (V9302), as well as the PD1/PDL1 blocker (BMS-1) [66]. Based on electrostatic interactions, the NH_2_ on V9302 and the pyrrole of Ce6 interacted with the were assembled by electrostatic interaction with the COOH on Ce6 and BMS. Also, hydrophobic interactions between aromatic and porphyrin moieties can facilitate assembly (Figure 3A). The BVC showed good drug delivery efficiency, robust photodynamic therapy (PDT) effect, and excellent ability to reprogram tumor metabolism. Therefore, BVC enhances immune recognition and reduces tumor cell immune escape to eradicate metastatic tumors (Figure 3B).

Similarly, Le and colleagues reported carrier-free NPs (IRE-NPs) using rapamycin (RAPA, chemotherapeutic drug), indocyanine green (ICG, photosensitizer) and protective polyphenols (EGCG) for synergistic chemo-photothermal therapy [67]. The hydrophobic and electrostatic interactions among RAPA, ICG, and EGCG molecules ensured the assembly of IRE-NPs. Compared with RAPA or ICG alone, IRE NPs demonstrated remarkable stability, photothermal effect, tumor accumulation ability, and anticancer efficacy in MCF-7 cells, HepG2 cells, and HeLa cells. The electrostatic and hydrophobic interactions can efficiently facilitate the assembly of carrier-free nanodrugs in water solution. Zhou and coworkers prepared a carrier-free self-delivery photothermal converter (CypCel) comprised of the photothermal agent Cypate (Cyp) and the anti-inflammatory drug Celecoxib (Cel) [68]. The Cyp and Cel with nonpolar groups could aggregate in aqueous solutions via Hydrophobic interactions. Besides, their polar groups mainly contacted water via electrostatic interactions, further promoting the assembly of CypCel. The CypCel could enhance tumor accumulation for superior photothermal therapy (PTT), and inhibit the inflammatory cascade against tumor cells.

### 2.4. Multiple Noncovalent Interactions

Since there are weak and highly unstable noncovalent interactions when acting alone, the aforementioned carrier-free nanodrugs mainly involve the co-driven assembly of hydrogen bonding, π-π stacking, electrostatic and hydrophobic interactions, respectively. These studies broaden the avenues to explore the interactions among noncovalent bonds. Moreover, the self-assembly process of carrier-free nanodrugs is also driven by triple-noncovalent bonds, which may play a role in advanced biological structures [55].

**Figure 4:**
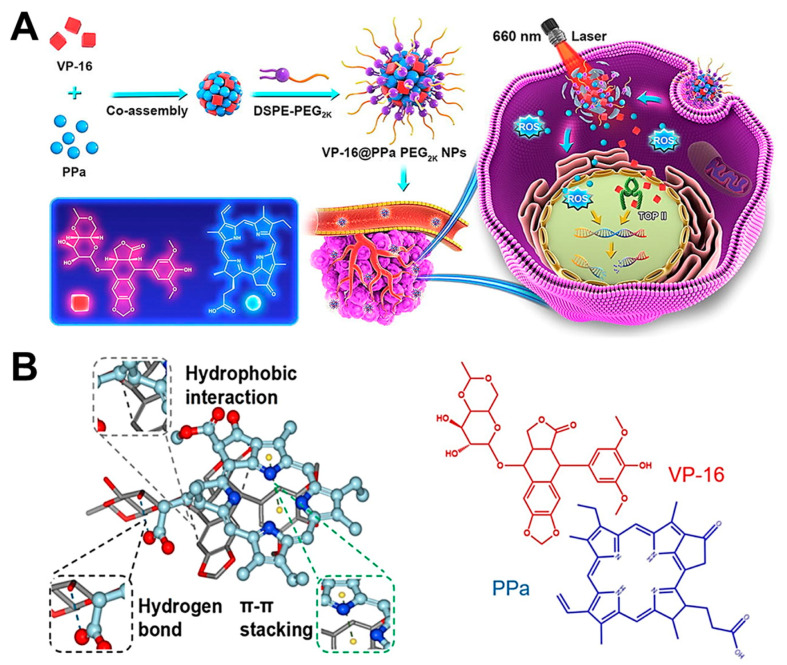
Schematic representation of VP-16@PPa co-assembly and DNA damage-mediated synergetic chemo-photodynamic therapy. (**A**) The VP-16@PPa PEG2K NPs was precisely delivered into tumor cells, multimodal DNA damage-mediated synergetic chemo-photodynamic therapy was realized under laser irradiation; (**B**) Molecular docking simulation of VP-16 and PPa. Reprinted with permission from ref. [69]. Copyright 2021, Elsevier.

As an illustration, Li et al. devised a hybrid carrier-free nanoassembly incorporating etoposide (VP-16, a topoisomerase II inhibitor), pyropheophorbide a (PPa, a photosensitizer), and a small amount of DSPE-PEG_2k_ (called VP-16@PPa PEG_2K_ NPs, Figure 4A) [69]. Macromolecular docking simulations showed that synergistic hydrophobic, π-π stacking, and hydrogen bonding interactions, facilitated the precise co-assembly of VP-16 and PPa (Figure 4B). The resulting nanoassembly showed high drug loading efficiency, prolonged blood circulation time, targeted accumulation within tumors, as well as a favorable antitumor effect in 4T1 breast cancer through multimodal DNA damage (Figure 4).

Carrier-free nanodrugs, directly co-assembled by chemotherapy drugs and NIR probes through multiple intermolecular noncovalent interactions, are promising alternatives to cancer hyperthermia. Chen et al. co-assembled hydrophobic zinc phthalocyanine (ZNPC, photosensitizer) and ICG to construct carrier-free nanodrugs (ZNPC-ICG) via triple noncovalent interactions. The ZNPC-ICG were then camouflaged with erythrocyte membrane to obtain ZNPC-ICG@RBC nanoprobes [70]. These carrier-free nanoprobes selectively accumulated in tumor cells and produced both ROS and hyperthermia under laser irradiation, thereby achieving remarkable tumor ablation without any regeneration by synergistic PDT and PTT. In general, the use of multifunctional antitumor agents as endogenous synergists to enhance chemotherapy-PDT is still a challenge in the design of carrier-free nanodrugs. Lan et al. developed GA-Ce6-FA NPs composed of gambogic acid (GA), Ce6, and folic acid (FA) via electrostatic, hydrophobic, and π-π stacking interactions [71]. The carrier-free NPs revealed performance in fluorescence imaging, specific tumor-targeting and chemotherapy-PDT synergistic antitumor efficacy.

Although all of the above carrier-free nanodrugs are self-assembled from non-covalent interactions, structural stability can be maintained during in vivo circulation. When nanoparticles enter the physiological environment, they may interact with large proteins, and their surface will be covered by a layer of protein, forming what is known as the protein “corona” [72]. For many NPs, the corona formation occurs so quickly that provides a steric stabilizing layer against aggregation by particle contact [73]. For example, citrate-stabilized gold NPs aggregate immediately in PBS, but are stable in plasma [74]. In addition, non-covalent forces within the nanoparticles can maintain the structural integrity of the NPs to some extent. This conclusion was proved by the researchers’ nanomedicine stability experiments in 10% fetal bovine serum (FBS) containing plasma proteins, peptides, fats, carbo-hydrates, growth factors, hormones, and inorganic substances [49,60].

To date, with the development of nanotechnology, surface modification of carrier-free nanodrugs has been accepted as a primary strategy to improve stability and reduce the non-specific adsorption of proteins in the blood. For example, the imparting of a hydrophilic polymer coating (e.g., PEG, proteins, DNA, bio-coatings, etc.) and biomimicry camouflage (e.g., red blood cells membrane) on the surface of carrier-free nanodrugs can reduce non-specific binding in vivo [67,75].

## 3. Covalent Bonds-Driven Carrier-Free Nanodrugs

Prodrugs, referring to inactive conjugates of drugs, possess biologically reversible modifications to achieve ideal physicochemical properties and are easily decomposed in vivo to release active drugs. [76,77]. Prodrugs can usually offer several virtues, such as improved drug solubility, high drug loading ability, prolonged drug circulation, reduced adverse effects, and tumor-specific drug release in response to stimulation [78,79]. However, due to their small molecular nature, prodrugs are still facing rapid degradation, premature activation, renal clearance, and hepatic metabolism in vivo [23]. To address these problems, prodrugs-based carrier-free nanoplatforms, integrating the prodrugs strategy and nanotechnology into one system, have been viewed as promising candidates for clinical translation [80].

Idealized prodrugs can be activated by specific stimuli in the tumor microenvironment (TME), such as acidity, high glutathione (GSH) or reactive oxygen species (ROS), and specific enzymes, for drug release on demand [81,82]. Therefore, rational design of prodrugs combined with nanotechnology for constructing carrier-free nanodrugs can significantly improve drug stability and pharmacokinetics, reduce renal clearance or degradation, extend drug circulation time, and enhance tumor accumulation via EPR effect and therapeutic efficacy [83,84,85].

Typically, amphiphilic prodrugs can be constructed by covalent bonds between hydrophobic and hydrophilic drugs, or drugs and functional biomolecules [86]. Taking advantage of intermolecular noncovalent interactions, these prodrugs are easily self-assembled into carrier-free nanodrugs. Importantly, they can co-assemble with or co-encapsulate additional drugs, biomolecules, and small prodrugs into carrier-free nanodrugs [27]. Based on the presence of many cleavable chemical bonds (e.g., acid-responsive imine /Schiff base bonds [28], reduction-responsive disulfide bonds [29], easily hydrolyzable ester bonds [16], and enzyme-cleavable specifically peptide linkers [30]), these prodrug-based carrier-free nanodrugs can realize controlled drug release in the special TME [87].

### 3.1. Imine Bond

Compared with the physiological pH 7.4, the tumor’s extracellular pH averages between pH 6.8 and pH 7.0, and even can get as low as 5.7 [88]. The transmembrane pH gradient varies in different subcellular components, such as mitochondria for pH 8.0, endosomes for pH 5.6–6.0, and lysosomes for pH 4.5–5.0 [89,90]. These microenvironmental differences provide prerequisites for regional management and targeting of pH-responsive carrier-free nanodrugs [91].

The imine (Schiff base) bond is a typical pH-responsive dynamic covalent chemical linkage, which is formed by the condensation reaction between aldehyde or ketone and amine [92]. The imine (Schiff base) bond is relatively stable at pH 7.4 but can be rapidly hydrolyzed under acidic conditions [93]. Thus, the imine (Schiff base) bond is a promising linker for designing pH-sensitive carrier-free nanodrugs due to its simple synthesis process and acid-labile properties.

**Figure 5:**
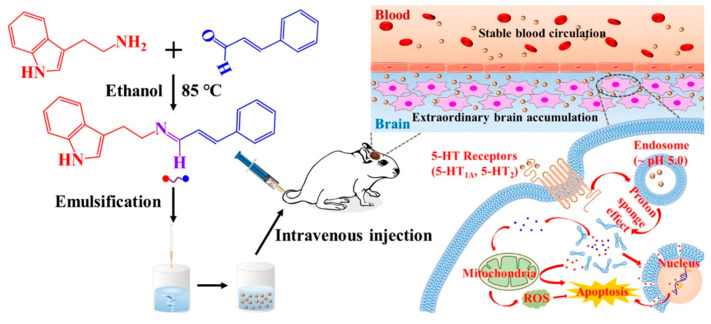
Schematic illustration of the self-assembly, in vivo transport and combinational antitumor therapy of the LD NDs. Reprinted with permission from ref. [94]. Copyright 2021, Elsevier.

This imine bond can be used to construct carrier-free nano-prodrugs derived from anticancer twin drugs. Wang et al. designed a hydrophobic tryptamine (Try, an analog of serotonin)-cinnamaldehyde (CA, promoting tumoral ROS-mediated apoptosis) twin drug by aldimine condensation, which further formed pH-triggered small molecule carrier-free nano-prodrugs (denoted as Try-CA-NPs (Figure 5) [94]. Try-CA-NPs showed circulation stability, as well as charge reversal, large-to-small size transition, and drug release under intracellular endosomal pH trigger. Furthermore, liposoluble Try-CA-NPs easily crossed the blood-brain barrier (BBB) and targeted glioma cells through Try-mediated cellular uptake, finally completely destroying SH-SY5Y multicellular spheroids.

Cytotoxic drugs (e.g., hydrophobic DOX) are commonly used in clinical cancer therapy at the maximum tolerated dose. Nevertheless, high doses of drugs often induce serious toxicity and drug resistance [95]. To achieve enhanced efficacy, hydrophobic drugs can be conjugated to other drugs or functional biomolecules via bioenvironment-responsive bonds, and then realize drug-controlled release at target sites. Fan et al. reported an amphiphilic drug-drug conjugate (LMWH-DOX) using hydrophilic low molecular weight heparin (LMWH) and DOX via imine bonds [96]. The LMWH-DOX conjugate is then self-assembled into carrier-free LD NDs, which enhances the tumor accumulation and releases the drugs under an acidic stimulus. Therefore, LD NDs exhibited significant inhibitory effects on tumor growth and metastasis with good biocompatibility.

Utilizing the acidic condition of the TME, Wu et al. first obtained fluorinated prodrugs DOX and melittin (MPI, cytolytic peptide) via Schiff base (imine) reaction, which denoted as FDOX (FD) and FMPI (FM), respectively [97]. Next, the FD, FM, and thymocyte selection-associated high mobility group box protein (TOX) were co-assembled into carrier-free FD/FM@siTOX NPs. These NPs showed potent pH-responsive drug release, tumor accumulation, anti-liver cancer immune response and metastasis.

### 3.2. Disulfide Bond

Tumor cells show higher oxidative and reducing stress states due to intracellularly overproduced ROS and GSH than normal cells, thus it is difficult to release parent drugs into the tumor [98]. Taking advantage of the redox gradient between tumor and normal cells, some specific chemical linkages can be utilized to design prodrugs which provide a prerequisite for constructing redox-responsive carrier-free nanodrugs [99]. Inspired by the chemical properties and functional effect, disulfide bonds are widely used as reduction-sensitive linkers in the design of antitumor prodrugs [85,100].

**Figure 6:**
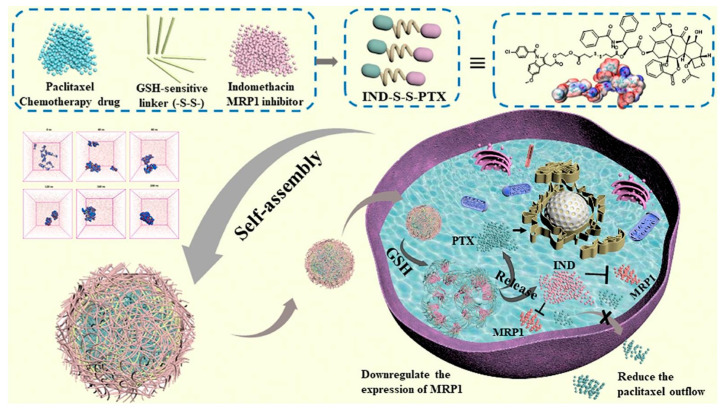
Schematic illustration of indomethacin-ss-paclitaxel nanodrugs for reversing multidrug resistance and enhancing NSCLC therapy. Reprinted with permission from ref. [103]. Copyright 2021, Elsevier.

When the disulfide bond is triggered by GSH, it is easy to convert into two -SH groups through a reduction reaction [101]. Connecting different drugs or functional biomolecules with disulfide bonds can fabricate multifunctional antitumor prodrugs-based carrier-free nanodrugs, achieving tumor-specific drug release. Pei and coworkers prepared a range of dimeric prodrugs by coupling two paclitaxel (PTX) molecules with different chain-length dicarboxylic acids via disulfide bonds. Each of these PTX dimers was able to self-assemble into NPs [102]. Furthermore, Kang et al. employed disulfide bonds to connect the indomethacin (IND, nonsteroidal anti-inflammatory drug) and PTX to obtain the drug conjugate, which self-assembled into carrier-free nanodrugs (IND-S-S-PTX NPs) by van der Waals force-driven self-assembly (Figure 6) [103]. The IND-S-S-PTX NPs realized PTX-controlled in response to high-level GSH in TME. The IC_50_ value of IND-S-S-PTX NPs was only about 1/3 of that of PTX, but the apoptotic ratio of IND-S-S-PTX NPs was almost five times that of PTX. Besides, the NPs could induce apoptosis towards A549/taxol and reverse multi-drug resistance by down-regulating MRP1 protein expression. However, when PTX was loaded into nanocluster carrier material, its anticancer activity was not significantly improved, which was almost the same as that of free PTX [104]. These studies show that connecting PTX with disulfide bonds and assembling prodrug-based carrier-free nanodrugs may significantly enhance the drug activity and antitumor potential than carrier delivery.

Apart from being widely used in the design of reduction-responsive carrier-free nanodrugs, the disulfide bond can also serve as an oxidation-responsive linker, because it is oxidized to sulfoxide or sulphone under oxidative stimulation [99]. Sun et al. reported three paclitaxel-citronellol (PTX-CIT) prodrugs linked by disulfide bonds-containing carbon chains with different lengths, which can be assembled into uniform NPs [101]. The disulfide bond-based prodrug NPs exhibited unique redox dual-responsiveness when exposed to co-stimuli of oxidation and reduction. The H_2_O_2_ oxidized disulfide bonds to hydrophilic sulfoxides or sulfones, facilitating the oxidation-responsive drug release and showing potent antitumor activity. This work provides the possibility for constructing redox dual-responsive carrier-free nanodrugs via disulfide bonds for cancer therapy.

The GSH is much higher in cancer cells than that in normal cells [105], thus carrier-free nanodrugs with disulfide bonds can add additional selective targeting for cancer treatment. Feng’s group developed a multifunctional carrier-free nano-photosensitizer (NPS) using one single amphiphilic glycosylated BODIPY (BSL) molecule containing two disulfide bonds [106]. For BSL NPS, the lactose moieties were hydrophilic targeting agents, while borondipyrromethenes (BODIPYs) were used as hydrophobic dual phototherapy (DPT) photosensitizers. BSL NPS showed superior biocompatibility, tumor-targetability, GSH-responsiveness, and precise release of BODIPY motif in TME, and simultaneously produced ROS and heat, which enhanced antitumor effect through synergistic PDT and PTT.

Additionally, disulfide bond-mediated prodrug nanoplatforms can be responsive to exogenous oxidants (single-linear oxygen, ^1^O_2_) induced by photosensitizers [107]. Yang et al. reported a prodrug nanoplatform encapsulating pyropheophorbide a (PPa) based on the disulfide bond bridged PTX and oleic acid (OA) [108]. The obtained prodrug nanosystem could respond to both redox heterogeneity in tumors and exogenous oxidants (singlet oxygen) produced by photosensitizers, then undergo the oxidative self-strengthened process and promoted PTX cascade release, realizing obvious chemo-photodynamic therapy against tumor.

### 3.3. Ester Bond

**Figure 7:**
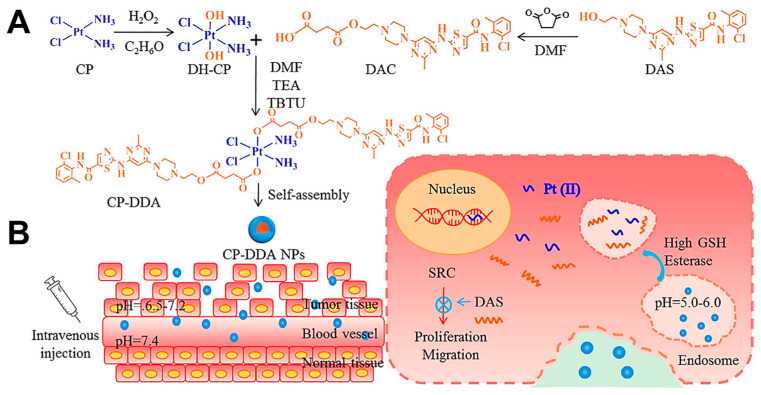
(**A**) The synthetic route of CP-DDA NPs; (**B**) Drug delivery mechanism of nanoparticles and drug release mechanism in tumor cells. Reprinted with permission from ref. [110]. Copyright 2021, Elsevier.

Ester bonds are commonly used for self-assemble of prodrugs because they can be decomposed by enzyme catalysis or spontaneous hydrolysis for drug release without specific stimulus [109]. The carboxylic ester linkage is an appealing option since many drugs or functional biomolecules contain carboxylic acid or alcohol functional groups [82]. Yang et al. developed an amphiphilic drug-drug conjugate (CP-DDA) using derivatives of dasatinib (DAS, hydrophobic drug) and cisplatin (CP, hydrophilic anti-tumor drug), which could further self-assemble into stable carrier-free nanoparticles (CP-DDA NPs, Figure 7) [110]. The DAS modified with succinic anhydride was bound to the CP derivative (DH-CP) via ester bonds (Figure 7A). After cellular uptake, CP-DDA NPs precisely released DAS and Pt(II) due to the hydrolysis of ester bonds and redox degradation of GSH in the tumor, thereby displaying potent synergistic antitumor activity and reduced toxicities of free drugs (Figure 7B).

In a different report, Li et al. used a succinic acid (SA) linker to connect the anticancer drug Ptx with the tumor-specific peptide RGD to form a Ptx-SA-RGD conjugate. It can be self-assembled into carrier-free nanofibres (P/T-NFs) with loading with tetrandrine (Tet, potential antitumor effects) [111]. These P/T-NFs improved the apoptosis-inducing effect via the ROS-initiated mitochondrial apoptotic pathway and showed a stronger antitumor effect.

Several ester bond-linked prodrugs carrier-free nanodrugs can exhibit pH-sensitive drug release characteristics. Fan et al. constructed an MTX-MAN conjugate by linking double-acting methotrexate (MTX, chemo-drug and FA receptors targeting ligand) and mannose (MAN, targeting ligand and chemo-drug for tumor therapy) via hydrolyzable ester bonds, which can self-assemble to from carrier-free MTX-MAN NPs [112]. MTX-MAN NPs could release drugs through double stimulus of lysosome acidity and esterase, and doubly recognized by tumor, exhibiting excellent tumor accumulation and synergistic chemotherapy effects.

### 3.4. Specifically Peptide Linker

Several enzymes are overexpressed during tumorigenesis and tumor metastasis, such as cathepsin B [113], and matrix metalloproteinases [114], which are important targets for DDS and therapeutics. Accordingly, these tumor-associated enzyme dysregulations could be exploited as endogenous triggers for designing enzyme-responsive prodrug carrier-free nanodrugs to achieve cancer diagnosis and treatment [79,88,115].

Peptide-drug conjugates (PDCs), one type of prodrug, are drug molecules or functional biomolecules that covalently attach to specific peptide sequences through enzymatically cleavable peptide linkers [116]. PDCs can increase the solubility of hydrophobic drugs, reduce toxicity, actively target specific receptors of tumor cells, and show enzyme-sensitive drug release and therapeutic properties of specific peptides [117]. Furthermore, PDCs can assemble to form carrier-free nanodrugs spontaneously or under certain stimuli [118]. Therefore, PDCs-based carrier-free nanodrugs can be employed for releasing therapeutic agents into tumors related to specific enzymes or enzymatic activity.

**Figure 8:**
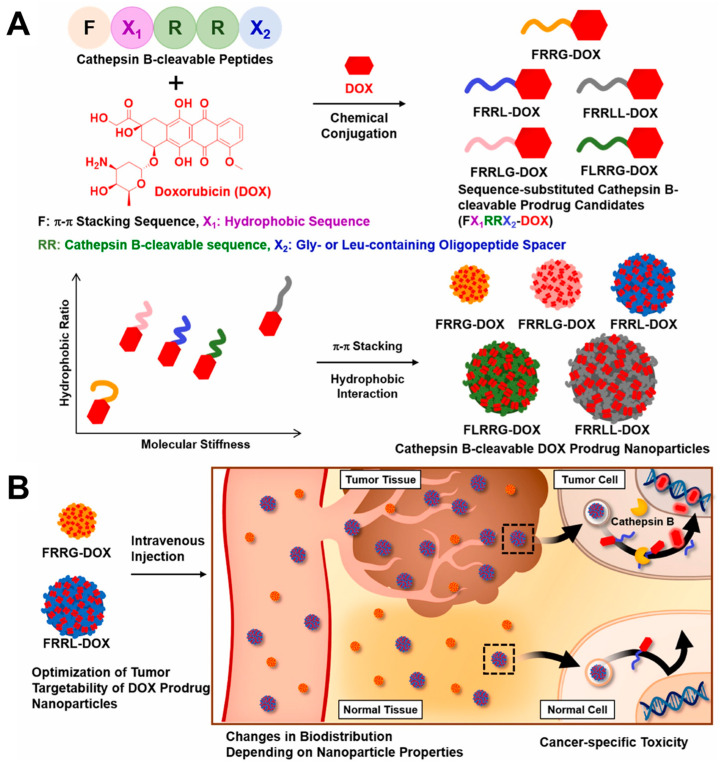
(**A**) Schematic synthetic procedure of VES-Ir prodrug and construction of self-assembled VES-Ir NPs, (**B**) co-delivery and low pH and esterase release and synergistic antitumor effect after endo-lysosomal internalization into tumor cell. Reprinted with permission from ref. [122]. Copyright 2022, Elsevier.

Cathepsin B is commonly overproduced in breast cancer, colorectal cancer, and prostate cancer. The targeted bio-enzyme of cathepsin B can serve as a tumor-specific biomarker, whilst it is also a protease that activates nano-prodrugs in tumors [119,120]. Using the pro-apoptotic peptide drug (SMAC), cathepsin B-cleavable peptide (FRRG), and DOX, Shim’s group developed a SMAC-FRRG-DOX conjugate, which self-assembled into DD-NPs [121]. The DD-NPs could be cleaved to release drugs by cathepsin B, displaying dual pro-apoptotic activity and chemotherapy for overcoming drug resistance. Subsequently, Shim et al. identified the most suitable peptide linker for cathepsin B-responsive DOX prodrug NPs [122]. Five peptide-DOX prodrugs were obtained by chemically coupling different cathepsin B cleavable peptides, *Phe-Arg-Arg-Gly* (FRRG), *Phe-Arg-Arg-Leu* (FRRL), *Phe-Arg-Arg-Gly* (FRRLG), *Phe-Leu-Arg-Leu-Gly* (FLRRG), and *Phe-Leu-Leu-Arg-Arg-Gly* (FLRRG), to DOX, all then self-assembled into carrier-free NPs (Figure 8A). Comparative studies revealed that FRRL-DOX nanoparticles showed uniform size, good stability, high cathepsin B-specificity, and efficient tumor cellular uptake. Besides, FRRL-DOX nanoparticles significantly reduced toxicity to normal tissues and induced tumor apoptosis (Figure 8B).

Matrix metalloproteinase-2 (MMP-2) is up-regulated in a wide range of tumors, so it can be used to design PDCs-based carrier-free nanodrugs [114]. Hu et al. prepared self-delivery MA-pepA-Ce6 NPs via bonding metformin (MET, PD-L1 inhibitor) to Ce6 through MMP-2 cleavable peptide (pepA) [123]. These MA-pepA-Ce6 NPs could realize effective drug accumulation under dual TME-specific stimuli sensitivity, demonstrating obvious antitumor immune response through the synergistic effect of PDT and PD-L1 inhibitor.

## 4. Metal Ions-Driven Carrier-Free Nanodrugs

The construction of carrier-free nanodrugs using noncovalent interactions (e.g., hydrogen bonding forces, hydrophobic and electrostatic interactions) is a facile synthesis process [124]. Nevertheless, the complex in vivo environment may lead to the decomposition of carrier-free nanodrugs according to the unstable nature of the noncovalent interactions, reducing drug efficacy and damaging normal tissues [125]. Although covalent linkages based-carrier-free nanodrugs are used during the systematic circulation, nanodrugs with excessively high stability may suffer from hindering drug release at the target site [33]. Consequently, it is necessary to develop therapeutic carrier-free nanodrugs through a simple, smart and versatile approach.

Recently, designing carrier-free nanodrugs by metal-coordinated self-assembly between metal ions and drug or functional biomolecules has gradually attracted great attention [22,126]. The coordinated covalent bonds exhibit stable and dynamic behavior in complicated environments since their strength is stronger than weak noncovalent interactions but weaker than the strong covalent bonds [127].

Metal ions show specific electronic, optical, radioactive, magnetic, and catalytic properties, and can integrate tumor diagnosis and therapy to achieve tremendous effect [128]. For example, Fe^2+^ and Cu^+^ can reduce H_2_O_2_ to produce highly toxic ROS for chemodynamic therapy (CDT) [129,130]. Mn^2+^ and Gd^3+^ can act as magnetic resonance imaging (MRI) contrast agents owing to their remarkable magnetic properties [131,132]. Therefore, metal ions can coordinate with various anticancer drugs, photosensitizers, or functional biomolecules to form carrier-free nanodrugs with versatile functionalities and synergetic therapy, which will provide more possibilities for effective tumor diagnosis and treatment. In this part, the metal ions-coordinated carrier-free nanodrugs will be discussed based on the extensive multifunction and application of metal ions in tumor diagnosis and treatment.

### 4.1. Fe^2+/3+^ Ions Coordination

Iron ions are the most commonly used coordination ions due to their excellent physicochemical and biological properties [133]. As an important active transitional metal element, ions usually participate in diverse intracellular activities, especially regulating the intracellular redox state. Fe^2+/3+^ ions induced Fenton reaction in CDT have been broadly investigated, which can exert Fenton activity under reducing and acidic TME, catalyzing H_2_O_2_ to produce ROS for killing tumor cells [33,134]. Apart from potential antitumor applications, compounds with ions can be also used as MRI contrast agents [135]. Moreover, Fe^2+/3+^ complexes have been shown to be excellent candidates for PTT [136]. Hence, Fe^2+/3+^-coordinated carrier-free nanodrugs are promising functional candidates for cancer therapeutics.

**Figure 9:**
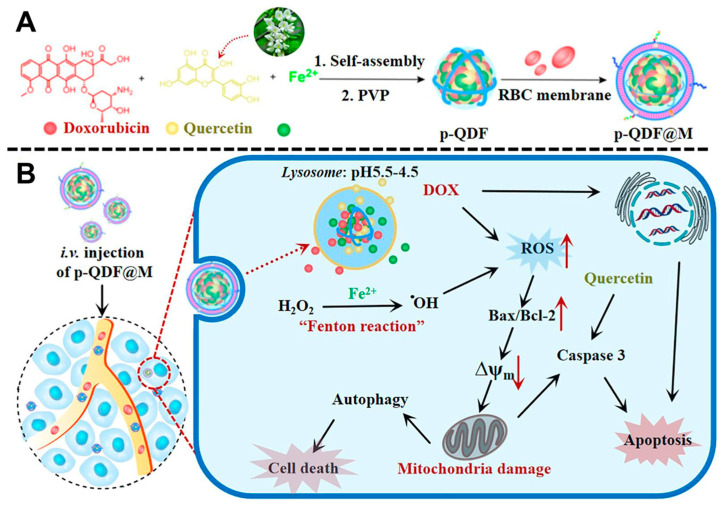
(**A**) The preparation process of p-QDF@M. (**B**) The biodistribution and mechanism of p-QDF@M for synergistic chemotherapy. Reprinted with permission from ref. [138]. Copyright 2021, Elsevier.

Iron ions can coordinate with natural polyphenols with multiple phenolic hydroxyl groups to form 3D metal polyphenol networks (MPN), which possess high specific surface areas and provide many adsorption sites for drug molecules [137]. Shang et al. reported Fe^2+^-coordinated carrier-free nanodrugs (p-QDF@M, Figure 9A) consisting of DOX, natural quercetin (enhancing pro-apoptotic signal), and RBC membranes for triple-negative breast cancer (TNBC) treatment [138]. The phenolic hydroxyl groups of quercetin could be coordinated with Fe^2+^ to form MPN. Then, DOX was effectively embedded into a 3D network of MPN via π-π stacking. After tumor cell internalization, the Fe^2+^ coordinated bonds in p-QDF@M were readily dissociated in lysosomal acidic conditions, thus releasing DOX and quercetin. Meanwhile, the released Fe^2+^ up-regulated the ROS level through the Fenton reaction, inducing mitochondrial damage and cancer cell apoptosis. Based on the synergistic effect of DOX, quercetin and Fe^2+^, p-QDF@M significantly enhanced the therapeutic effect against TNBC and inhibited pulmonary metastasis (Figure 9B).

The coordination forces between ions and multiple drugs can maintain stability, avoid premature drug leakage, and enhance therapeutic outcomes. As a typical example, Fan et al. designed carrier-free Trojan-horse metal-organic nanotheranostics with reducible diameters by coordinating-assembly of pemetrexed (PEM), Fe^III^ ions, and antiangiogenesis pseudolaric acid B (PAB) [139]. These nanotheranostics were stable in physiological conditions but transformed into ultra-small-size nanotheranostics under moderately acidic TME, which were self-target internalized into the tumor. Additionally, these nanodiagnostics achieved visualization of self-targeting ability through computed tomography/magnetic resonance imaging, displaying excellent antitumor and antiangiogenic effects.

ROS, generated from the Fe^2+^-induced Fenton reaction, can also react with other substances, producing more toxic components for killing tumor cells. Chen and coworkers reported a redox-triggered C-centered free radicals nanogenerator, denoted as Fe(III)-ART NPs, via coordination self-assembly of Fe^3+^ and hydrolyzed artemisinin (ART) [140]. In a high GSH environment, the carrier-free Fe(III)-ART NPs released ART and Fe^3+^. Subsequently, Fe^3+^ was further reduced to Fe^2+^, which catalyzes the endoperoxide of ART to generate CDT. The Fe(III)-ART NPs not only realized self-enhanced magnetic resonance imaging but also presented excellent CDT effects.

### 4.2. Cu^2+^ Ions Coordination

Apart from Fe^2+/3+^ ions, various other transition metal ions can also exhibit analogous Fenton-like reactions to kill tumor cells. Copper, as an essential trace element in humans, has been demonstrated to be another primarily dominant coordinated ion, because its catalytic reactivity is more energetically competitive than that of other transition metal ions [141]. Cu^2+^ ions can be transformed into Cu^+^ ions through the interaction with intracellular GSH within the TME. Concurrently, the GSH is converted to GSSH, which protects the generated ROS and induces apoptosis of tumor cells [142]. Meanwhile, Cu^+^ catalyzes an efficient Fenton-like reaction that spontaneously converts H_2_O_2_ to highly toxic ·OH and other reactive oxygen species in weak acidic and neutral conditions [143]. Copper ions with the ability to deplete GSH and produce ·OH have become promising agents for tumor therapy in CDT [144,145].

**Figure 10:**
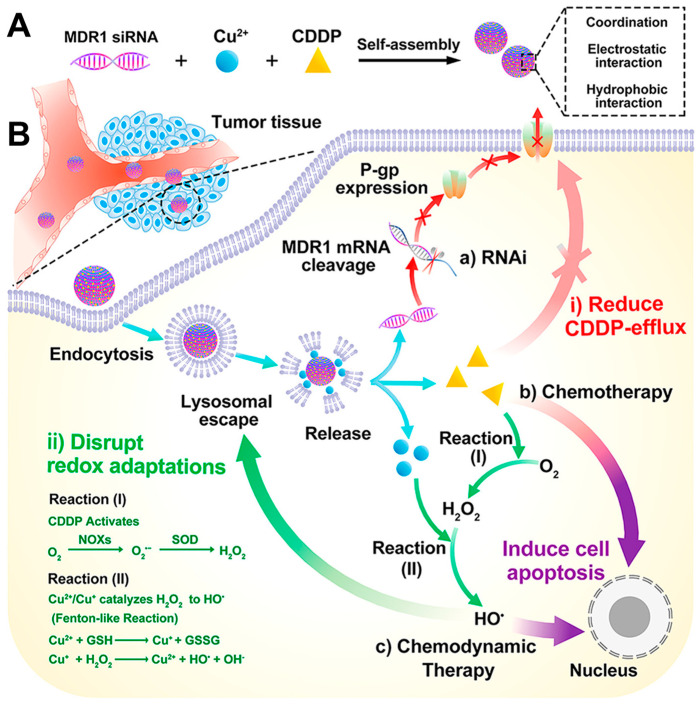
(**A**) Preparation of Cu-siMDR-CDDP. (**B**) Mechanisms of Cu-siMDR-CDDP in overcoming drug resistance. Reprinted with permission from ref. [130]. Copyright 2021, American Chemical Society.

Recently, Jiang et al. developed a carrier-free platform (Figure 10A) by assembling Cu^2+^, the chemotherapeutic cisplatin (CDDP), and multidrug resistance protein 1 (MDR1) siRNA [130]. The Cu-siMDR-CDDP system could respond to release CDDP, Cu^2+^, and siRNA in the acidic TME. This triggered cascade bioreactions for producing H_2_O_2_. Subsequently, Cu^2+^-induced Fenton-like reactions converted H_2_O_2_ into HO^•^ with GSH depletion, thereby disrupting the redox adaptation mechanisms in drug-resistant tumors. Additionally, the MDR1 siRNA inhibited the expression of P-glycoprotein (P-gp) and CDDP efflux (Figure 10B). Such Cu-siMDR-CDDP showed good therapy in inhibiting tumor growth and overcoming drug resistance.

Cu^2+^ ions can enhance the photothermal conversion efficiency and ROS productivity of a single photosensitizer. In view of the superior coordination capacity between two sulfonate anions of ICG and Cu^2+^ ions, Chen et al. designed the carrier-free PhA@NanoICG nanotheranostic through the coordination between Cu^2+^ and ICG, whilst encapsulating photodynamic-agent pheophorbide A (PhA) [146]. After PhA@NanoICG is internalized inside the tumor, Cu^2+^ ions could improve the photothermal conversion efficiency of ICG, induce GSH depletion, and destroy the cellular antioxidant defense system, thereby avoiding ROS consumption and improving the phototherapeutic outcomes.

### 4.3. Mn^2+^ Ions Coordination

Mn ion is not only a trace metal element closely related to human physiological function but also a common paramagnetic metal ion, thereby regularly serving as an MRI contrast agent for guiding cancer treatment [147]. Mn^2+^ ions display superior GSH removal ability to break the redox homeostasis of TME, this is conducive for ROS-based PDT and CDT [148]. Also, Mn^2+^ can catalyze the decomposition of endogenous H_2_O_2_ into O_2_, thus carrier-free nanodrugs formed by Mn^2+^ coordination can be used for cancer PDT ignoring the limitation of hypoxic TME [149]. Based on these findings, Mn^2+^ can coordinate with various drugs or functional biomolecules (e.g., anticancer drugs and photosensitizers [150] or photothermal agents [151]) to engineer carrier-free nanodrugs for imaging-guided tumor therapy.

**Figure 11:**
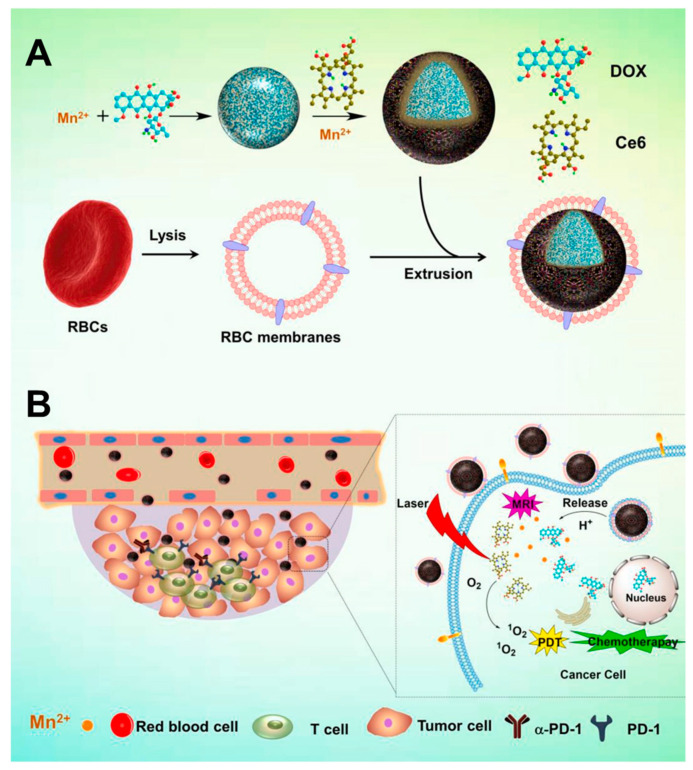
(**A**) Preparation of red blood cell membrane-coated “multi-in-one” core-shell metal ion-drug nanoparticles. (**B**) Schematic of the combination therapy in the tumor microenvironment. Reprinted with permission from ref. [152]. Copyright 2021, Elsevier.

For instance, Geng and coworkers revealed a core-shell nanoplatform by Mn^2+^-coordinated self-assembly (Figure 11A) [152]. Mn^2+^ coordinated with DOX and Ce6 to generate the core (chemotherapy) and the shell (PDT), respectively. Upon cloaking with RBC membranes, the “multi-in-one” nanodrugs exhibited improved IC_50_ value (0.116 μg/mL), prolonged circulation within the blood and enhanced accumulation at tumor sites. Owing to the Mn^2+^ coordination, these Mn^2+^-drug nanoparticles showed limited drug leakage in normal tissues but rapidly decomposed to release the drug in the acidic TME, thus demonstrating MRI-guided enhanced chemical-PDT therapy (Figure 11B). In a typical carrier delivery comparison example, Zheng et al. used hierarchical core-shell nanoparticles to encapsulate DOX (ZnO-DOX@ZIF-8), and the drug loading capacity was only 11.2%. In vitro, antitumor results showed that the cell survival rate only decreased to about 50% at a concentration of 3.125 μg/mL (0.4 μg/mL for free DOX) [153]. In comparison, the Mn^2+^ coordinated carrier-free assembly strategy effectively enhanced the DOX drug activity.

In a recent report, Liu’s group designed dual-photosensitizers co-loading nanodrugs (IMCP) using the Mn^II/III^, ICG, and Ce6 via Mn^II^-coordination co-assembly [131]. The Mn^II^ presented in the IMCP exhibited the ability to deplete GSH within tumor cells, thus facilitating ROS-based PDT therapy with good biosafety in the dark. Additionally, IMCP integrated the inherent fluorescence, the photoacoustic imaging capability of photosensitizers, and the MRI contrast potential of Mn^II/III^. This amalgamation conferred remarkable imaging abilities for tumor diagnostics, achieving excellent tumor accumulation, self-enhanced phototherapy, and the guidance of multi-modal imaging for PDT/PTT-mediated tumor eradication.

Furthermore, the coordination between Mn^2+^ and the porphyrin ring within the TME following MnO_2_ degradation is also advantageous for PDT. Chu and colleagues introduced an innovative O_2_ self-sufficient nanodrug delivery platform, defined as MnO_2_/DVDMS. This platform was reduced by GSH and H_2_O_2_ to produce Mn^2+^ and O_2_. Subsequently, the Mn^2+^ could coordinate sinoporphyrin sodium (DVDMS, photosensitizer) to form nanotheranostics (nanoDVD) in situ, which improved the PDT efficacy. The elevated stacking of porphyrins within the nanoDVD enhanced the PTT effect [154]. This tumor environment-triggered NanoDVD enabled multimodal magnetic resonance, fluorescence, and photoacoustic imaging guidance, including for effective cancer treatment.

### 4.4. Gd^3+^ Ions Coordination

Magnetic gadolinium (Gd) is a widely used MRI contrast agent approved by the U.S. Food and Drug Administration (FDA), and it readily coordinates with O or N to form chelates [155]. Moreover, as the coordination number decreases, the longitudinal relaxation (*r*_1_) of Gd^3+^ ions will increase, this can improve its imaging ability in TME [156].

**Figure 12:**
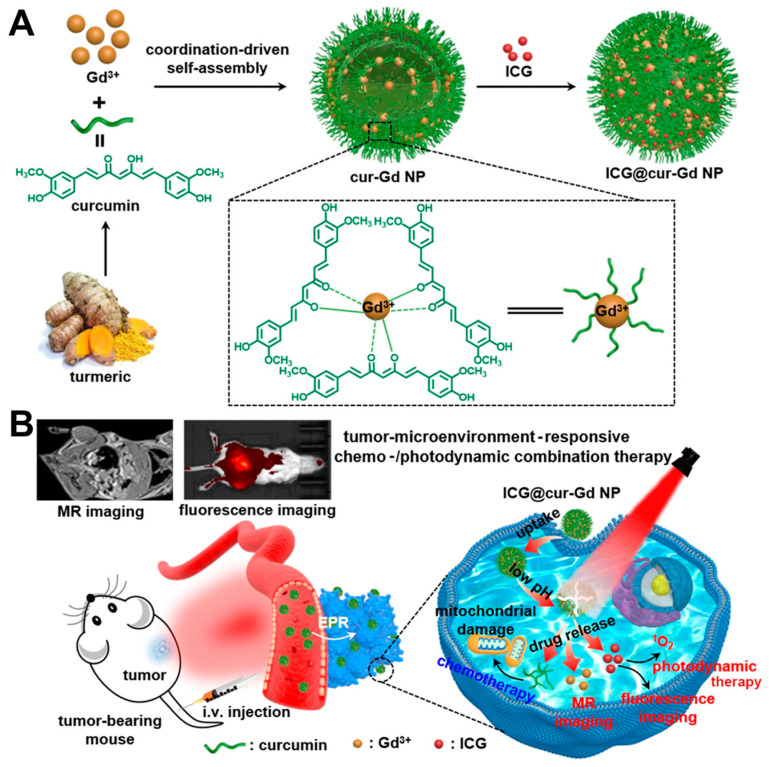
(**A**) Preparation of tumor acid-responsive ICG@cur-Gd NPs (**B**) Schematic representation of ICG@cur-Gd NPs in imaging-guided cancer therapy. Reprinted with permission from ref. [132]. Copyright 2021, American Chemical Society.

In this aspect, Wen et al. successfully developed an ICG-loaded carrier-free MPN theranostic agent through the coordination between curcumin and Gd^3+^, denoted as ICG@cur-Gd NPs (Figure 12A) [132]. Compared with the curcumin loaded in the spherical polymer nanostructures (2.755% of drug loading capacity) for combinatorial tumor therapy [157], the ICG@cur-Gd NPs assembled by the coordination of curcumin and Gd3+ significantly improve the bioavailability and drug loading of curcumin. The tumor acidic microenvironment induced the decomposition of the theranostic agent to specifically release ICG, curcumin, and Gd^3+^, thereby greatly enhancing the chemo-/photodynamic therapeutic efficacy with good systemic biocompatibility. Dual magnetic resonance (MR)/fluorescence imaging of ICG@cur-Gd NPs presented precise tumor visualization for guiding synergistic chemo-photodynamic cancer treatment (Figure 12B).

In another study, Fan and coworkers used methotrexate (MTX, anticancer and targeting agent), Gd, and artesunate (ASA, CDT-like agent) to coordinately assemble into carrier-free ASA-MTX-Gd^III^ NPs [158]. Following self-targeting to cancer cells through the FA receptor, this nanoplatform underwent decomposition triggered by lysosomal acidity, releasing Gd^III^ ions, ASA, and MTX. Then, the endogenous Fe^II^ ions, overproduced specifically from the tumor, reacted with ASA to generate ROS that acted synergistically with MTX. With MRI guidance and self-targeting, ASA-MTX-Gd^III^ NPs showed coincident and synergetic chemodynamic-chemotherapy, resulting in ROS amplification and the near complete eradication of the tumor.

Moreover, Gd, as a high-Z element, holds promising for sensitizing radiation therapy (RT) [159]. Based on this characteristic, Huang and associates engineered carrier-free nanorods (ZGd-NR) through Gd^3+^ coordinating with zoledronic acid (Zol) [160]. The ZGd-NR utilized deposited X-rays to generate enough ·OH, which induced robust immunogenic cell death (ICD). This process also involved the consumption of tumor-associated macrophages and suppression of regulatory cytokines. Meanwhile, the released Zol caused apoptotic cell death of tumor-based macrophages by inhibiting the mevalonate pathway and reprograming TME. Therefore, ZGd-NR-sensitized RT not only promoted immune activation but also improved the efficacy of checkpoint blockade immunotherapy.

### 4.5. Zn^2+^ Ions Coordination

As another essential element, Zn shows excellent biocompatibility within the human body and holds a vital role in ensuring specific functions in the body [33]. Currently, the carrier-free nanostructures based on Zn coordination primarily involve the coordinated interaction between Zn and N atoms on imidazole and porphyrin rings [161], as well as amino acid/peptide/protein [162]. Hence, the assembly driven by Zn^2+^ ion coordination is a promising direction in the integration of drugs or functional biomolecules to construct innovative carrier-free tumor theranostic nanosystems.

As a typical example, Zhong’s group developed a zinc-coordinated carrier-free nanodrug (PZB NP) via the coordination among protoporphyrin IX (PpIX), Zn^2+^, GSH inhibitor _L_-butyrate sulfoximine (BSO) [161]. The multifunctional PZB NP could reduce existing intracellular GSH before PDT, and further block the production of antioxidant enzymes after PDT, thus amplifying ROS-induced tumor cell damage in a two-pronged manner and enhancing the antitumor PDT efficacy.

During the self-assembly process of carrier-free nano-prodrug, coupling hydrophobic drugs with natural amino acids and utilizing Zn^2+^ coordination-driven multimolecular interactions is an effective strategy. Xu et al. reported a carrier-free nano-prodrug (AHZ) by the coordination interaction between hydrophobic artesunate-histidine conjugate (AH) and Zn^2+^, in which histidine possessed strong coordination affinity with Zn^2+^ [31]. This AHZ nano-prodrug was quickly disassembled in the TME with weak acidity and high GSH levels and showed better anticancer performance than direct administration, which is suitable for cancer targeting.

Zn^2+^ ions can participate as cofactors in the coordination assembly of carrier-free nanodrugs. Shi’s team prepared amphiphilic DNAzyme-TBD conjugate by grafting AIE photosensitizer (TBD-Br) with a phosphorothioated DNAzyme skeleton, then self-assembled into nanoparticles, accompanied by the surface phosphorothioate groups coordinated with Zn^2+^ to obtain hybrid carrier-free DNAzyme NPs [163]. Because the surface Zn^2+^ provided sufficient cofactor supply, and TBD-Br triggered ^1^O_2_ production upon light, the obtained DNAzyme NPs effectively induced tumor cell apoptosis. Additionally, they also down-regulated early growth response factor-1 protein (EGR-1), leading to the inhibition of tumor cell proliferation.

### 4.6. Other Metal Ions Coordination

Aside from the abovementioned metal ions, several other metal ions are gradually used to construct carrier-free nanodrugs for cancer theragnostic applications. For instance, gold (Au)-based carrier-free nanodrugs have attracted increasing interest in the field of targeted delivery, bioimaging, theranostic and biosensing. Wang et al. synthesized the Au(III)-tetra-(4-pyridyl)porphyrin (AuTPyP) through the coordination of Au(III) and porphyrin, the AuTPyP monomers were further self-assembled into carrier-free Au-based porphyrin nanorods (AuPNSs) with heat/acid dual stimulation-responsiveness [164]. The resultant AuPNSs possessed excellent photo- and heat-conversion efficiencies for PTT, facilitating hydrophobic pyridine group protonation and subsequently drug release in acidic TME. With cRGD modification, cRGD-AuPNSs significantly enhanced cellular uptake and synergistic chemo-photothermal therapy efficiency.

To date, many significant achievements have been reported by the coordinated-driven self-assembly of Pt metallacycles and metallacages [165,166,167,168,169]. Platinum ions can form coordination bonds with numerous ligands, enabling the preparation of carrier-free nanodrugs for cancer theranostics [167]. As a typical example, Fu et al. fabricated carrier-free platinum (IV) methylene blue (MB) coordination nanotheranostics, which further encapsulated 10-hydroxycamptothecin (CPT) and represented as CPM [168]. The porous shuttle-shape CPM effectively catalyzed endogenous H_2_O_2_ into O_2_ for PDT enhancement. Furthermore, the red-light irradiation combined lysosomal acidity co-triggered the CPT release from CPM, enhancing localized production of mitochondrial ROS. This synergistic strategy achieved effective tumor ablation through phototherapy. In another work, Xing and coworkers reported a carrier-free self-delivery nanodrug via platinum (II)-tolfenamic acid (tolf, a selective COX-2 inhibitor) conjugate (Tolfplatin) containing coordination bonds, which could assemble into NPs [169]. Compared with cisplatin, Tolfplatin NPs significantly inhibited the growth and metastasis of cancer, endowing cisplatin with stronger anticancer efficacy.

Calcium ions (Ca^2+^) are another prominent macroscopic element in the human body, existing in almost all mammalian cells and playing vital roles in many cellular processes, including proliferation, metabolism and death [170]. As reported, tumor cells are sensitive to Ca^2+^, and intracellular calcium overload is associated with malignant tumors [171]. Calcium overload can induce apoptosis or programmed necrotic apoptosis by causing mitochondrial dysfunction to increase ROS production, rather than a specific pathway of cell death [172,173]. Presently, Ca^2+^ ions can be employed to construct self-assembled nanostructures under specific physiological and pathological conditions with pH, enzyme, and redox stimuli, resulting in tumor-specific and long-term therapies. Qin and colleagues introduced a carrier-free nanovaccine based on a calcium-bisphosphonate coordination system. The nanovaccine was designed for the co-delivery of bisphosphonates (BPs), tumor antigens, and lipid A (a TLR4 agonist) [174]. The incorporated BPs demonstrated the ability to activate dendritic cells (DCs) and innate-like γδT cells. In addition, the nanovaccine exhibited notable antigen presentation and induced targeted lysis of tumor cells. Furthermore, nanovaccines in combination with PD-1 antibodies enhance antitumor immunity. Besides, Huang et al. revealed an ACaT carrier-free nanodrug composed of alendronate, cyclin-dependent kinase 7 (CDK7) THZ1 and Ca^2+^ [175]. Ca^2+^ and alendronate formed the network structure through coordination interaction, then THZ1 was self-assembled into the structure via hydrophobic attraction. THZ1 caused calcium overload by coordinating with excessive Ca^2+^ and increased intracellular ROS production, synergistically disrupting calcium homeostasis and triggering cancer cell apoptosis. Such ACaT carrier-free nanodrugs could specifically target tumors, achieve antimigration and promote cell apoptosis in intraperitoneal disseminated ovarian cancer.

In short, metal ions-coordinated carrier-free nanodrugs used for cancer theranostic have the following advantages: (ⅰ) Metal ions with specific functions confer a variety of theranostic capabilities for carrier-free nanodrugs. (ⅱ) Metal ions-coordinated carrier-free nanodrugs display outstanding stability, drug-loading efficiency, EPR effect and biosafety. (ⅲ) Metal ions-coordinated nanodrugs effectively improve targeting efficiency, thus enhancing their accumulation in the tumor. (ⅳ) Metal ions-coordinated carrier-free nanodrugs specifically respond to the acidic TME in tumors and disrupt the TME.

## 5. Drug Nanocrystals

Drug nanocrystals (NCs) are nanoscale drug crystals (100–1000 nm), which are usually composed of active drugs and/or a small amount of surfactants to maintain their stability [176]. To date, the preparation methods of NCs mainly involve wet-ball milling, high-pressure homogenization methods, and nanoprecipitation [177,178]. Additionally, the combination techniques of precipitation, spray-drying, freeze-drying, wet-ball milling, and then annealing by high-pressure homogenization can further maintain the stability of the crystals [179]. Typically, NCs possess simple and green preparation, high drug loading capacity (nearly 100%), good structural stability, prolonged circulation time, and improved therapy effective in vivo, making them a promising candidate for the next generation of nanomedicine for clinical cancer and other diseases (e.g., infectious diseases) treatment [180,181].

In the past few years, many hydrophobic anticancer drugs, such as camptothecin (CPT), hydroxycamptothecin (HCPT), and paclitaxel (PTX), have been formulated as NCs for cancer chemotherapy [182]. Compared with their corresponding free drugs, these NCs exhibit considerable or even better tumor cell internalization and anticancer effects [183]. Yang et al. prepared 10-HCPT NCs by acid-base microprecipitation combined with high-pressure homogenization, which showed higher drug accumulation and better anti-cancer efficacy in 4T1 cells than that of 10-HCPT solution [184]. Moreover, 10-HCPT NCs also showed enhanced anti-proliferative effects on MCF-7, A549, HepG2, and HeLa tumor cells.

The fabrication of multiple drugs into drug nanocrystals allows for tumor synergistic treatment, which is a promising strategy for improving therapy efficiency. For instance, Zhang and coworkers utilized strong intermolecular interactions between PTX and cyclooxygenase-2 inhibitor indomethacin (IDM) to engineer IDM-induced PTX nanocrystal aggregates [185]. The IDM/PTX assemblies could transform into smaller nanoparticles and target tumor sites through the EPR effect in vivo. Compared with free PTX or IDM and PTX mixtures, IDM/PTX assemblies exhibited significantly enhanced antitumor effects based on synergistic immunotherapy and chemotherapy.

However, some NCs undergo poor drug targeting, low tumor accumulation rate, and shorter drug half-lives in vivo due to the lack of surface protection, resulting in poor cycling effects [177]. To effectively deliver antitumor drugs, controlling the cycling stability and surface performance of NCs through surface modification is of great significance. For example, Wang et al. developed DSPE-PEG 2000 modified PTX nanocrystals (PNCs) [180], which exhibited long-term stability, sustained release, higher area under curve (AUC), and lower clearance rate than bare PNCs. As another example, Su et al. reported anti-CD11 b antibody (Ab)-coated PTX NC (Ab/PTX NC), which could specifically target activated neutrophils. Then, the neutrophils effectively took up Ab/PTX NC and mediated their delivery to tumor tissue through the tumor vascular barrier, enhancing mouse survival in both preclinical lung cancer and glioma models [186].

## 6. Summary and Perspectives

The development and application exploration of smart carrier-free nanodrugs is a meaningful research orientation in the field of nano-biomedicine. Compared with inorganic/organic nanocarrier-based nanomedicines, carrier-free nanodrugs display impressive features, simple composition, flexible fabrication, high drug-loading efficiency, and avoidance of the toxicity of the carrier itself, which greatly endows them with clinical antitumor potential in the future. We outlined the assembly mechanisms behind carrier-free nanodrugs based on drugs or functional biomolecules with diverse chemical structures or properties, which will help researchers grasp the distinguishing features, self-assembly behaviors, and construction rules of various carrier-free nanodrugs.

Although great progress has been achieved, there are still some scientific issues and challenges of carrier-free nanodrugs that need to draw attention for clinical application in the future:

(1) It is difficult to control active drug ratios precisely. Since drugs or functional biomolecules with various structures have an impact on their interactions during assembly, the actual resulting carrier-free nanodrugs are distinct from the original feed ratio. In particular, multiple drugs/functional biomolecules are co-assembled by strong and specific interaction forces, leading to difficulties in changing the proportion of drugs and accurately controlling the morphology, particle size, and surface charge of carrier-free nanodrugs [13]. Generally, precise screening of dose ratios will maximize the antitumor effect. Therefore, developing a hopeful strategy for the designation of carrier-free nanodrugs with accurate control of drug proportion is crucial.
(2) Insufficient targeting efficiency. Most carrier-free nanodrugs enter the tumor mainly through the EPR effect, which is not enough to achieve high tumor selectivity. Recently, multifunctional nanodrugs with surface-modified targeting ligands (e.g., antibody, aptamer, peptide) have shown outstanding specific targeting ability toward cancer cells. Nevertheless, the targeting capacity is achieved through incorporating additional active targeted agents. Accordingly, exploring carrier-free nanodrugs with exceptional tumor-specific self-targeting deserves further studies.
(3) The lack of a good understanding of pharmacokinetics in vivo. It is essential to have in-depth knowledge of the pharmacokinetic behaviors and destiny of carrier-free nanodrugs in the human body before clinical application. Although numerous reports have shown that carrier-free nanodrugs perform better in prolonging circulation time, enhancing cellar uptake and tumor accumulation, and avoiding rapid clearance, the mechanism of drug metabolism and pharmacological action in vivo should be deeply investigated to promote their clinical application.
(4) The rationality of drug compatibility has not been well illustrated. Generally, there is antagonism, no interaction, additivity, or synergism between drug molecules. How to select the best optimal drug combination according to the synergistic effect and intermolecular interactions between drug molecules still lacks reliable research [187,188]. Thus, it is urgent to design carrier-free nanodrugs through reasonable drug compatibility for exerting the prime synergistic therapeutic efficiency, reducing potential metabolic risks and toxic-side effects.
(5) The limited therapeutic applications. To date, although carrier-free nanodrugs can act as excellent nano-candidates, their applications mainly involve cancer therapy. There are few carrier-free nanodrugs designed for the treatment of bacterial infections or other diseases. We look forward to these advanced performances of carrier-free nanodrugs in cancer treatment, including PDT, PTT, CDT, RT, fluorescence imaging, and MRI, can be extended to antibacterial fields, which will undoubtedly bring innovative prospects for antibacterial [189,190] anti-inflammatory [191,192], antiviral [193], wound healing [194] and other fields.
(6) The lack of deeper research. The exploration and study of carrier-free nanodrugs still stand in their infancy. The stability of long-term preservation needs to be fully considered. The potential toxicity to organs as well as the body’s immune response triggered by carrier-free nanodrugs have received little attention. Furthermore, large-scale industrial production and application in clinical practice are still big challenges to all the carrier-free nanodrugs. It needs to make more great efforts to conduct intensive research on carrier-free nanodrugs for clinical applications.

Overall, carrier-free nanodrugs have shown significant advantages in cancer therapy and present an expansive perspective for drug delivery systems, which offer enormous potential for advantageous clinical antitumor outcomes. Despite this, there is a long way for carrier-free nanodrugs to transfer from laboratory investigation to practical clinical translation, people are firmly convinced that these carrier-free nanodrugs will open up an innovative and wide avenue for the diagnosis and therapy of clinical diseases.

## Data Availability

This study did not report any data.
